# Factors Associated With Knowledge Sharing Among Health Sector Employees in Riyadh, Saudi Arabia

**DOI:** 10.7759/cureus.90996

**Published:** 2025-08-25

**Authors:** Fahad Al Mofeez, Anwar Al Enazi, Abdurahman Alzomia, Aeyd Al Qarni, Abdulrhman Alkhalf, Ahmed Alharthi, Sahar Alamoudi, Turky Ateeq, Habis Alanazi, Noura Alenezi, Nouf Alanazi, Muna Hassanein

**Affiliations:** 1 Training and Institutional Development, Branch of the Ministry of Health, Riyadh Region, Riyadh, SAU; 2 Compliance, Branch of the Ministry of Health, Riyadh Region, Riyadh, SAU; 3 Central Naseem Health Center, Second Health Cluster, Riyadh Region, Riyadh, SAU; 4 Northern Naseem Health Center, Second Health Cluster, Riyadh Region, Riyadh, SAU

**Keywords:** individual factors, knowledge collecting, knowledge donating, knowledge sharing, organizational factors, technology use factors

## Abstract

Introduction

Understanding the factors that influence the knowledge-sharing process among the health sector employees is essential for improving service quality and organizational performance. The aim of this study was to examine the relationship between knowledge sharing, including knowledge donating and knowledge collecting, and individual, organizational, and technological factors among healthcare employees in the Riyadh region of Saudi Arabia.

Methods

An analytical, cross-sectional study was conducted among 264 healthcare professionals and administrative staff in the healthcare sector selected through convenience sampling. Data were collected using an online questionnaire covering variables on knowledge sharing (dependent variable), and individual factors, organizational factors, and technology use factors (independent variables). Structural equation modeling was applied to investigate the relationships between the dependent and independent variables.

Results

Both the individual factors and the technology use factors were associated with knowledge donating and collecting. Individual factors had standardized path coefficients of 0.175 and 0.425 for knowledge donating and collecting, respectively (p-values < .001). Similarly, technology use factors showed coefficients of 0.411 (p-value =0.012) and 0.723 for knowledge donating and collecting, respectively (p-value < .001). Management support was not associated with either knowledge donating or knowledge collecting, with coefficients of 0.342 and -0.276, respectively (p > .05).

Conclusion

Individual and technology use factors were found to be associated with knowledge sharing, whereas organizational factors showed no significant association. The findings offer insight into how health sector employers could foster a knowledge-sharing culture to improve service quality.

## Introduction

Knowledge has become a cornerstone of the global economy, serving as an essential resource in organizational services, with the success of societies and economies depending on their ability to effectively manage and utilize this valuable asset [1,2]. Knowledge management (KM) is defined as the systemic and organizationally specified process of creating, acquiring, capturing, sharing, and using knowledge to enhance performance in organizations [2-4].

Knowledge sharing (KS) is an essential component in knowledge management and is a key element in systems aimed at enhancing performance [1,5]. At the organizational level, knowledge sharing can be defined as capturing, organizing, reusing, and transferring experience-based knowledge within the organization and making it available to all employees [6,7]. It is designed to transform individual knowledge into organizational knowledge [7]. It occurs between individuals regarding ideas, suggestions, work-related expertise and experience, values, and information and skills [6,8,9]. Other terms that similarly describe knowledge sharing include knowledge transfer, knowledge diffusion, knowledge distribution, and information sharing [8].

Knowledge sharing consists of two sub-behaviors: knowledge donating and knowledge collecting. Knowledge donating refers to the act of transferring one's personal intellectual capital to others [6,9]. It involves an employee’s willingness to actively communicate with colleagues [6,7,9]. On the other hand, knowledge collecting is the process of communicating with and encouraging others to share their knowledge or intellectual capital [6,9]. It includes actively consulting with colleagues to learn from them [6,7,9].

Organizations emphasize knowledge sharing as a way to foster knowledge exchange and creation among employees, which in turn enhances their capacity for innovation [6].

Knowledge management and sharing have been widely studied across multiple disciplines, including the healthcare sector [1], with increasing emphasis on recognizing knowledge as a critical asset for healthcare organizations [4]. Healthcare organizations are, by nature, highly knowledge-intensive, engaging professionals from various disciplines who must continuously keep pace with evolving technologies and specialized expertise [10,11]. Given that the healthcare sector has a direct impact on individuals' health, well-being, and quality of life, there is a strong emphasis on providing high-quality and efficient care [10]. To achieve this, healthcare providers must access accurate, up-to-date information to expand their knowledge and offer evidence-based services [4]. Knowledge sharing plays a vital role in this context, as it facilitates access to information and resources that support continuous learning, effective problem-solving, skill enhancement, and staying updated in the field [2].

Although knowledge sharing across various health systems is recognized worldwide, its implementation remains limited [5]. Knowledge sharing is influenced by numerous factors, generally categorized into individual, organizational, and technology-related elements [6,8,9,12,13]. At the individual level, crucial influences include enjoyment in helping others and an individual's knowledge self-efficacy [7-9,12,13]. Organizational factors impacting knowledge sharing encompass top management support and the presence and nature of reward systems [8,9,12-14]. Finally, technology-related factors, such as the effective utilization of information and communication technology (ICT), the usability of knowledge management systems, and the support provided by platforms like enterprise social media, are vital in enabling and facilitating these processes [3,8,9,14-16]. These diverse elements collectively determine the effectiveness of knowledge sharing.

In Saudi Arabia, several studies across higher education, industrial, commercial, education, and telecommunications sectors have investigated the determinants of knowledge sharing within organizations, emphasizing key aspects such as cultural characteristics [15], organizational learning and self-worth [16], and various individual-level factors [17-19]. This study builds on that body of research by focusing on the healthcare sector, where knowledge sharing is essential for improving service quality and organizational performance. The aim of this study was to examine the relationship between knowledge sharing, encompassing both knowledge donating and knowledge collecting, and individual, organizational, and technological factors among healthcare employees in the Riyadh region of Saudi Arabia. Specifically, the study aimed to assess the association between knowledge sharing and individual factors, including enjoyment in helping others and knowledge self-efficacy; organizational factors, represented by top management support and organizational rewards; and the use of ICT.

## Materials and methods

Study design and settings

This analytical, cross-sectional study was carried out in the Riyadh Region, where healthcare delivery is coordinated by the Regional Branch of the Ministry of Health (MoH). Services in the region are offered through an extensive network of facilities operated by various entities, including health clusters, non-MoH government sectors, and private providers. Across these facilities, healthcare professionals, including physicians, pharmacists, nurses, and support staff, are engaged in delivering care. Further, administrative functions, such as human resources, finance, logistics, and information technology, are provided by administrative staff at the MoH’s regional office, the three health clusters, and other governmental bodies operating outside the MoH.

Study population and sampling

The inclusion criteria for the study population encompassed healthcare professionals and administrative staff employed in both governmental and private healthcare sectors within the Riyadh Region, representing both genders and all nationalities, including physicians, dentists, pharmacists, nurses, and support staff. Exclusion criteria included individuals employed outside the health sector or outside the Riyadh Region.

A convenience sampling method was employed, consistent with approaches used in comparable studies [6,8]. Participants were recruited using two approaches. For employees of the regional Ministry of Health branch, the questionnaire link was distributed via official emails through the internal communication department. For employees in health clusters, non-MoH government sectors, and private sector, WhatsApp Messenger was used as the primary recruitment platform. WhatsApp is the most widely used social media application in Saudi Arabia [20] and is frequently employed for professional communication within the healthcare system [21]. The research team distributed the survey link within healthcare professional WhatsApp groups and invited members to participate. Participants were also encouraged to share the link with other healthcare professional groups, applying a network snowball sampling approach that leverages social connections to recruit additional participants and maximize coverage of the target population in this non-list-based, non-probability sampling method [22].

The required sample size was estimated using OpenEpi Version 3 (SSPropor), assuming a population of 10,000, a 90% confidence level, and a 5% margin of error, resulting in a calculated sample size of 264 eligible participants.

The instrument and data collection

Data were collected using a self-administered, structured, and pre-coded questionnaire consisting of two sections (see Appendix 1). The first section covered background information, including socio-demographic variables (e.g., age, gender, marital status) and job-related variables (e.g., qualifications, occupational category). The second section included questions on knowledge donating, knowledge collecting, and factors influencing the knowledge-sharing process, including individual factors, organizational factors, and technology use factors. Items for these constructs were adapted from previous studies that employed the same components [6,8,23]. Individual factors were measured by two sub-components: enjoyment in helping others, assessed through two items reflecting the pleasure employees derive from sharing knowledge; and knowledge self-efficacy, evaluated using four items assessing confidence in sharing valuable knowledge. Organizational factors were measured by two sub-components: top management support, captured with four items reflecting perceived encouragement for knowledge sharing from senior leadership; and organizational rewards, assessed with four items measuring employees’ beliefs about receiving incentives, such as bonuses, promotions, or job security, for sharing knowledge. Technology use factors were measured by ICT use, evaluated with four items assessing the usability and capability of technology for knowledge sharing. The outcome variables included knowledge donating, measured with three items assessing willingness to share knowledge with colleagues, and knowledge collecting, measured with four items reflecting practices related to learning from others. All the items were measured on a four-point Likert-type scale (1 = strongly disagree, 4 = strongly agree). The items adapted from previous studies were translated into Arabic and back-translated into English. The questionnaire was pilot-tested with a sample of 15 employees from non-health sectors to evaluate the clarity and comprehensibility of the items. The questionnaire was developed electronically using Google Forms and activated prior to dissemination to the target population. The first section of the questionnaire included an informed consent statement, in which potential respondents were asked whether they agreed to participate in the study. They were given two options: ‘Agree’ or ‘Do not agree.’ If ‘Do not agree’ was selected, the questionnaire automatically ended and was submitted. If ‘Agree’ was selected, it was considered an electronic signature, indicating consent to proceed with the remainder of the questionnaire. The research team monitored the survey process to ensure the quality and completeness of responses. They also sent reminder notifications through both channels one week after the initial invitation to minimize self-selection bias, encourage completion, and promote further dissemination within WhatsApp groups. The questionnaire was deactivated once the target sample size was achieved. All data were transmitted directly to a password-protected database, with access limited to authorized research personnel

Data analysis

Data were analyzed using IBM SPSS Statistics, version 21.0 (IBM Corp., Armonk, NY, USA) and jamovi version 2.6.44 (https://www.jamovi.org). Descriptive statistics were used to summarize background data in the form of frequencies and percentages. For the purpose of the analysis, three composite variables were created: the individual factors category, the organizational factors category, and the technology use factors category. Each category was computed by summing the scores of the items within its respective group. Reliability of the measurement scales was assessed using Cronbach’s alpha. Correlation analysis was conducted to evaluate the validity of the constructs. The measurement model included five latent variables: knowledge donating (three indicators), knowledge gathering (four indicators), individual factors (enjoyment in helping others and self-efficacy indicators), organizational factors (top management support and reward system indicators), and technology use factors (four ICT use indicators). Factor loadings, thresholds for ordinal variables, and residual variances were estimated. We identified four items exhibiting high inter-item correlations exceeding 0.2 and standardized regression weights (β) below 0.6. To enhance the model fit and reduce redundancy, these items were removed (two items from the self-efficacy construct and two items from the managerial rewards construct). Model fit was assessed using multiple fit indices: chi-square statistic, Root Mean Square Error of Approximation (RMSEA) with 95% confidence interval, Standardized Root Mean Square Residual (SRMR), Comparative Fit Index (CFI), Tucker-Lewis Index (TLI), Bentler-Bonett Non-Normed Fit Index (NNFI), Bollen’s Relative Fit Index (RFI), Incremental Fit Index (IFI), and Parsimony Normed Fit Index (PNFI). The Hoelter’s critical N was calculated at the 0.05 significance level. Structural Equation Modeling (SEM) was then performed to test the hypothesized paths between the independent variables (individual factors, organizational factors, and technology use factors) and the dependent variables (knowledge donating and knowledge gathering). All latent constructs were allowed to correlate.

The study was approved by the Institutional Review Board of Princess Nourah bint Abdulrahman University (IRB Log Number: 25-0141 - March 5, 2025).

## Results

The study included 264 participants, and their background information is presented in Table 1. Regarding sociodemographic characteristics, 128 (48.5%) were under 40 years of age, 197 (74.5%) were male, and 194 (73.3%) were married. Concerning educational background and occupation, 145 (54.9%) held undergraduate degrees, 126 (47.9%) were paramedic staff, 80 (30.3%) were physicians, dentists, or pharmacists, and 58 (21.8%) were administrative staff. The majority of participants (81.1%) were employed in the public healthcare sector, while only 18.9% worked in the private sector.

**Table 1:** Background information of the study participants (N= 264)

	Frequency	Percent
Age		
< 40 years	128	48.5
41-60 years	95	36.0
> 60 years	41	15.5
Gender		
Male	197	74.5
Female	67	25.5
Marital status		
Married	194	73.3
Single	41	15.6
Divorced	18	6.9
Widowed	11	4.2
Qualifications		
Undergraduate level	145	54.9
Postgraduate level	119	45.1
Occupational Category		
Administrative staff	58	21.8
Paramedic staff	126	47.9
Physician / dentist / pharmacist	80	30.3
Sector		
Public	214	81.1
Private	50	18,9

Structural model

A reliability test was conducted to assess the internal consistency among items measuring each construct. The Cronbach’s alpha was 0.776 for individual factors, 0.806 for organizational factors, 0.745 for technology use factors, 0.684 for data donating, and 0.752 for data collecting. Correlation analysis revealed that most item pairs had correlation coefficients ranging from 0.3 to 0.7, indicating moderate to strong associations and supporting good convergent validity. However, the items ‘I share my skills with colleagues when they ask for it’ and ‘Colleagues in my company share knowledge with me when I ask them to’ in the data collecting construct exhibited a lower correlation coefficient of 0.280.

**Figure 1:**
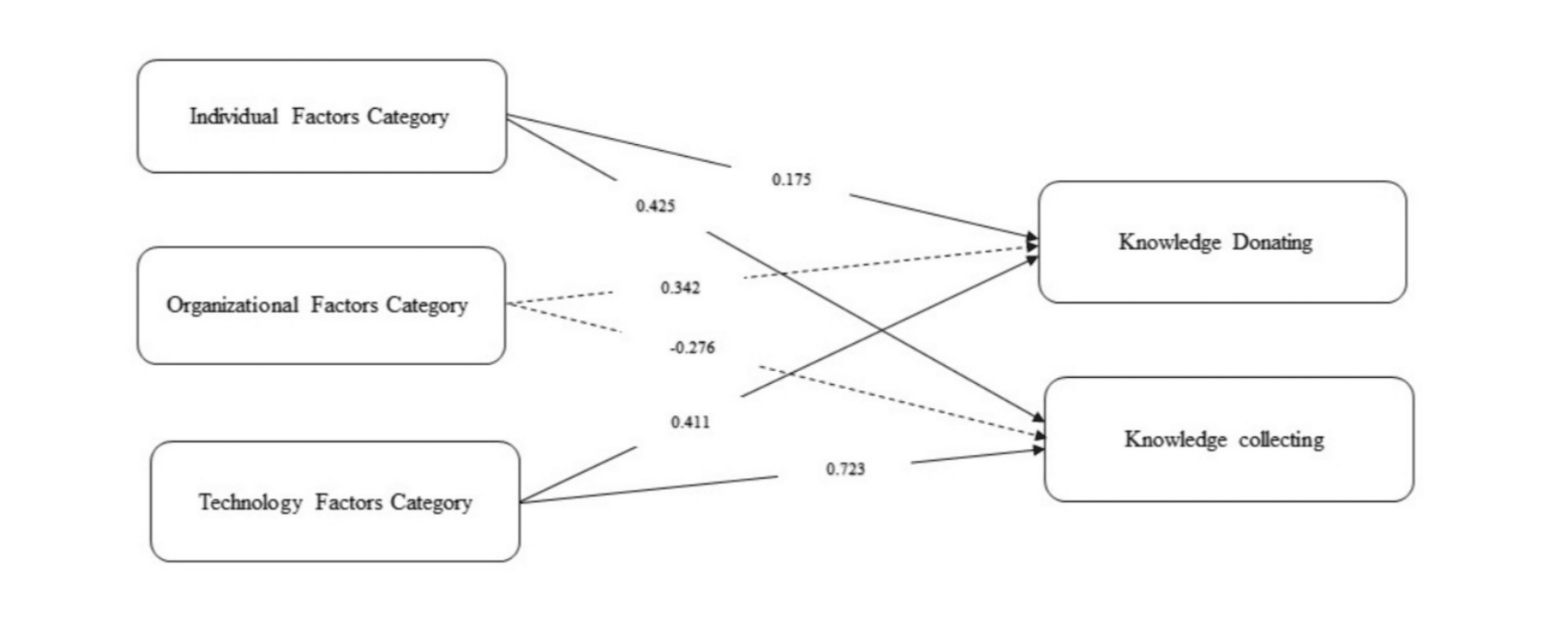
Results of structural equation model

The initial stage of model estimation involved assessing the hypothesized model’s goodness-of-fit. Following this, the significance of each proposed path within the research model was evaluated. Figure 1 presents the results, with bold lines indicating significant paths and dashed lines representing non-significant ones. 

The SEM analysis demonstrated an acceptable model fit, with a chi-square value of 572 (df = 199, p < .001), a CFI of 0.981, a TLI of 0.978, a NNFI of 0.978, and a RFI of 0.966, RMSEA of 0.072, and a SRMR at 0.066, suggesting the model was a good fit for the data (Table 2).

**Table 2:** Model Fit Summary (Structural Equation Modeling Analysis)

Index	Observed value
chi-square (χ²)	572
Comparative Fit Index (CFI)	0.981
Tucker-Lewis Index (TLI)	0.978
Bentler-Bonett Non-normed Fit Index (NNFI)	0.978
Bollen's Relative Fit Index (RFI)	0.966
the Root Mean Square Error of Approximation (RMSEA)	0.072
Standardized Root Mean Square Residual (SRMR)	0.066

Structural equation model

Both individual factors and technology use factors emerged as associated with knowledge donating and knowledge collecting. The individual factors category, which includes the variables enjoyment in helping others and self-efficacy, showed standardized path coefficients of 0.175 and 0.425 for knowledge donating and knowledge collecting, respectively (p-values < .001). Similarly, the technology use factors category demonstrated standardized path coefficients of 0.411 (p-value = 0.012) and 0.723 (p-value<0.001) for knowledge donating and knowledge collecting, respectively (p-values < 0.05) (Table 3, Figure 1). In contrast, management support was not associated with knowledge sharing, with standardized path coefficients of 0.342 and -0.276 for knowledge donating and knowledge collecting, respectively (p-values > 0.05) (Table 3, Figure 1).

**Table 3:** Association of Independent Variables with Knowledge donating (Structural Equation Modeling Path Estimates) P-values < 0.05 are considered statistically significant. Significant p-values are indicated with an asterisk (*)

Dependent	Predictors	Path coefficient	β	z	P-value
Knowledge donating	Individual factors category	0.175	0.214	3.64	< 0.001
Knowledge donating	Organizational Factors category	0.342	0.372	1.93	0.053
Knowledge donating	Technology use factors category	0.411	0.457	2.53	0.012
Knowledge collecting	Individual factors category	0.425	0.493	6.16	< 0.001
Knowledge collecting	Organizational Factors category	-0.276	-0.284	-1.23	0.217
Knowledge collecting	Technology use factors category	0.723	0.76	3.39	< 0.001

## Discussion

The aim of this study was to assess the relationship between the categories of individual factors, organizational factors, and technology use factors and knowledge sharing, as represented by knowledge donating and knowledge collecting. The results indicate that both individual factors and technology use factors were associated with these two sub-behaviors of the knowledge sharing process. In contrast, the organizational factors category was not significantly associated with either knowledge donating or knowledge collecting.

Our results show that the individual factors category was strongly associated with individuals’ willingness to share their knowledge, which is consistent with results from other studies. The enjoyment in helping others, a component of individual factors, is consistently recognized as a significant and powerful driver of knowledge sharing [7-9]. It is often considered the most important factor impacting the frequency of knowledge sharing [9]. Individuals are intrinsically motivated to contribute knowledge because they find intellectual pursuits pleasurable or challenging, and they enjoy helping others, which is positively related to knowledge-sharing attitudes and intentions [7,9]. Similarly, previous studies concluded that knowledge self-efficacy, another component of individual factors, positively influenced knowledge sharing [14,24]. Employees who are confident in their knowledge are generally more likely to both donate and collect it [24]. A previous study in the Saudi Arabian higher education sector, although using different components of the individual factors category, also reported a significant effect on knowledge-sharing attitude [17]. Saudi Arabia scores high on collectivism in Hofstede’s cultural dimensions, reflecting a strong orientation toward group cohesion and loyalty [25]. This collectivist orientation strongly influences attitudes toward knowledge sharing [26]. Therefore, a practical implication for employers is to enhance employees’ enjoyment by fostering positive mood states related to social exchange and by stimulating intrinsic motivation for knowledge sharing [8]. This can be achieved through meaningful feedback and targeted training, which help build confidence and promote knowledge-sharing behaviors [8,24].

Our results also confirmed those of previous studies regarding the positive association between technology use and knowledge sharing. ICT use is identified as a significant factor influencing knowledge sharing processes, positively impacting both knowledge donating and knowledge collecting [8]. Previous studies conducted in Saudi Arabia's higher education institutions, as well as in the industrial and commercial sectors, have also reported significant effects of technology use on knowledge-sharing attitudes [19-21]. ICT is considered a critical enabler and foundational component of a Knowledge Management plan, simplifying the processes of knowledge capture, creation, transfer, and reuse [3], as they facilitate various KM activities, including information creation, identification, acquisition, organization, distribution, and retrieval [3]. They are crucial for overcoming geographical limitations and time barriers in knowledge transfer, enabling communication and collaboration among dispersed teams [3,7]. ICT also plays a significant role in knowledge reuse, enabling rapid search, access, delivery, and retrieval of electronically stored information [3]. Therefore, employers should ensure that employees receive adequate training for effective ICT use [7], in addition to investing in ICT infrastructure, to enhance knowledge sharing and ultimately improve organizational performance [14].

Our results showed no association between knowledge sharing and organizational factors represented by top management support and organizational rewards in the form of financial incentives and job promotion. These results do not agree with most of the previous studies that consistently identified organizational factors as a significant factor that positively influences knowledge sharing processes [8,14], including studies conducted among employees of education, higher educations, and telecommunications sectors in Saudi Arabia [15]. However, our results should be interpreted with caution, given the use of convenience sampling, the cross-sectional design, and reliance on self-reported data. Nevertheless, our findings may reflect the organizational culture in Saudi Arabia, which is characterized by high power distance. This cultural context fosters an environment where knowledge primarily flows from the top down, with limited mechanisms or incentives for subordinates to contribute, question, or openly share information and ideas with their superiors or across hierarchical levels [25]. In addition, the relationship between management support and knowledge sharing was found to be conditional rather than direct, operating through mediating factors such as trust and affiliation [27] and job interdependence [28]. Future research should explicitly investigate these mediating mechanisms to clarify their role and to develop more effective strategies for enhancing knowledge sharing.

This study has several limitations. The use of a convenience sampling approach may have introduced selection bias, resulting in a non-representative sample of health sector employees, which reduces external validity and limits the generalizability of the findings both within Saudi Arabia and to other settings [22]. The snowballing component further constrained our ability to define the exact sampling frame, determine the number of healthcare employees invited, calculate a response rate, or conduct a comprehensive response analysis. Nevertheless, the results provide valuable insights to guide future research and inform targeted interventions within the Riyadh health sector. Another limitation is the use of a four-point Likert scale, which reduces measurement sensitivity compared to five- or seven-point alternatives. The neutral option was intentionally excluded to minimize responses driven by fatigue or time pressure, particularly relevant for health sector employees, as this could have diluted the discriminative power of the data [29]. Moreover, given that the topic is not obscure, inclusion of a neutral option might have encouraged socially desirable responses [30]. A further limitation is reliance on self-reported data, which may be subject to social desirability bias, as respondents could overstate knowledge-sharing behaviors to present themselves favorably. Such bias is known to distort self-reports and threaten data validity. However, self-report remains widely used in the literature, with similar studies adopting this approach due to its practicality and efficiency. Finally, the cross-sectional design limits the ability to establish causal relationships. Nonetheless, this design has been applied in similar studies and remains appropriate for the exploratory aims of the present research.

## Conclusions

In conclusion, both individual factors and technology use factors were associated with knowledge sharing among employees in the health sector. Understanding the influence of individual motivation and technology use provides valuable direction for organizations seeking to implement targeted strategies that strengthen knowledge exchange. These results underscore the importance of fostering positive mood states associated with social exchange and stimulating intrinsic motivation through meaningful feedback and targeted training. Additionally, organizations should provide adequate training in the effective use of ICT and invest in ICT infrastructure to support and enhance knowledge-sharing behaviors. In contrast, management support was not found to be associated with knowledge sharing, a result that should be interpreted with caution given the study’s limitations and the cultural context of Saudi Arabia.

## Appendices

Appendix 1 

**Table 4:** Questionnaire

Factors Associated with Knowledge Sharing Among Health Sector Employees in Riyadh, Saudi Arabia					
Questionnaire					
Please answer these questions by selecting the appropriate answer					
	Question			Options	
	Age				
	Gender			Male Female	
	Marital Status			Married Single Divorced Widowed	
	Educational Qualification			Bachelor's/Diploma Postgraduate Diploma Master's Doctorate/PhD	
	Employee Classification			Administrator Non-physician health practitioner Doctor/Dentist/Pharmacist	
	Years of Experience in Current Position/Job				
	Years of Total Experience				
	Sector			Government Health Private Health	
Choose the answer that suits you					
	enjoyment in helping others				
	- I enjoy sharing my knowledge with colleagues			Strongly disagree Disagree Agree Strongly agree	
	- I enjoy helping colleagues by sharing my knowledge			Strongly disagree Disagree Agree Strongly agree	
	knowledge self-efficacy.				
	- I am confident in my ability to provide knowledge that others in my company find valuable			Strongly disagree Disagree Agree Strongly agree	
	- I have the expertise to provide knowledge that is valuable to my company			Strongly disagree Disagree Agree Strongly agree	
	- It doesn't really matter whether I share my knowledge with colleagues or not (reverse coding)			Strongly disagree Disagree Agree Strongly agree	
	- Most other employees can provide more valuable knowledge than I can (reverse coding)			Strongly disagree Disagree Agree Strongly agree	
	top management support				
	- Senior managers believe that encouraging knowledge sharing with colleagues is beneficial			Strongly disagree Disagree Agree Strongly agree	
	- Senior managers always support and encourage employees to share their knowledge with colleagues			Strongly disagree Disagree Agree Strongly agree	
	- Senior managers provide the necessary assistance and resources to enable employees to share knowledge			Strongly disagree Disagree Agree Strongly agree	
	- Senior managers are keen to ensure that employees are satisfied with sharing their knowledge with colleagues			Strongly disagree Disagree Agree Strongly agree	
	Your organization is capturing new knowledge that contributes to improving work			Strongly disagree Disagree Agree Strongly agree	
	Organizational rewards				
	- Sharing my knowledge with colleagues should be rewarded with a higher salary			Strongly disagree Disagree Agree Strongly agree	
	- Sharing my knowledge with colleagues should be recognized with a reward			Strongly disagree Disagree Agree Strongly agree	
	- Sharing my knowledge with colleagues should be rewarded with a promotion			Strongly disagree Disagree Agree Strongly agree	
	- Sharing my knowledge with colleagues should be rewarded with increased job security			Strongly disagree Disagree Agree Strongly agree	
	information and communication technology use				
	- Employees extensively use electronic storage (such as online databases and data warehouses) to access knowledge			Strongly disagree Disagree Agree Strongly agree	
	- Employees use knowledge networks (such as groupware, intranets, virtual communities, etc.) to communicate with colleagues			Strongly disagree Disagree Agree Strongly agree	
	- Our organization uses technology that allows employees to share knowledge with other people within our organization			Strongly disagree Disagree Agree Strongly agree	
	- Our organization uses technology that allows employees to share knowledge with other people outside our organization			Strongly disagree Disagree Agree Strongly agree	
	Donate knowledge				
	- When I learn something new, I tell my colleagues about it			Strongly disagree Disagree Agree Strongly agree	
	- When my colleagues learn something new, they tell me about it			Strongly disagree Disagree Agree Strongly agree	
	- Sharing knowledge among colleagues is normal in our organization			Strongly disagree Disagree Agree Strongly agree	
	Gather knowledge				
	- I share my knowledge with colleagues when they ask for it			Strongly disagree Disagree Agree Strongly agree	
	- I share my skills with colleagues when they ask for it			Strongly disagree Disagree Agree Strongly agree	
	- Colleagues share knowledge with me when I ask them to			Strongly disagree Disagree Agree Strongly agree	
	- My colleagues in my organization share their skills with me when I ask them to			Strongly disagree Disagree Agree Strongly agree	
	- The organization's ability to innovate				
	- Our organization frequently experiments with new ideas			Strongly disagree Disagree Agree Strongly agree	
	- Our organization seeks new ways of doing things			Strongly disagree Disagree Agree Strongly agree	
	- Our organization is innovative in working methods			Strongly disagree Disagree Agree Strongly agree	
	- Our organization is often the first to introduce new services			Strongly disagree Disagree Agree Strongly agree	
	Benefit from knowledge transfer				
	Knowledge transfer contributed to improving my knowledge and skills			Strongly disagree Disagree Agree Strongly agree	
	Knowledge transfer contributed to improving my performance			Strongly disagree Disagree Agree Strongly agree	
